# Human Placental-Derived Adherent Stromal Cells Co-Induced with TNF-α and IFN-γ Inhibit Triple-Negative Breast Cancer in Nude Mouse Xenograft Models

**DOI:** 10.1038/s41598-017-18428-1

**Published:** 2018-01-12

**Authors:** Hoshea Allen, Niva Shraga-Heled, Michal Blumenfeld, Tamar Dego-Ashto, Dana Fuchs-Telem, Ariel Gilert, Zami Aberman, Racheli Ofir

**Affiliations:** Pluristem Ltd., Haifa, 31905 Israel

## Abstract

Culturing 3D-expanded human placental-derived adherent stromal cells (ASCs) in the presence of tumor necrosis factor-alpha (TNF-α) and interferon-gamma (IFN-γ) transiently upregulated the secretion of numerous anti-proliferative, anti-angiogenic and pro-inflammatory cytokines. In a 3D-spheroid screening assay, conditioned medium from these induced-ASCs inhibited proliferation of cancer cell lines, including triple-negative breast cancer (TNBC) lines. *In vitro* co-culture studies of induced-ASCs with MDA-MB-231 human breast carcinoma cells, a model representing TNBC, supports a mechanism involving immunomodulation and angiogenesis inhibition. *In vivo* studies in nude mice showed that intramuscular administration of induced-ASCs halted MDA-MB-231 cell proliferation, and inhibited tumor progression and vascularization. Thirty percent of treated mice experienced complete tumor remission. Murine serum concentrations of the tumor-supporting cytokines Interleukin-6 (IL-6), Vascular endothelial growth factor (VEGF) and Granulocyte-colony stimulating factor (G-CSF) were lowered to naïve levels. A somatic mutation analysis identified numerous genes which could be screened in patients to increase a positive therapeutic outcome. Taken together, these results show that targeted changes in the secretion profile of ASCs may improve their therapeutic potential.

## Introduction

Despite progress in developing targeted therapies for certain breast cancer subtypes, since triple-negative breast cancers (TNBC) lack estrogen receptor (ER) and progesterone receptor (PR) and do not over-express the human epidermal growth factor receptor 2 (HER2), they are not amenable to current therapies that target those receptors. TNBC accounts for approximately 15% of all breast cancer cases, and the only current options for treatment are a combination of non-specific therapies, i.e. chemotherapy, surgery and radiation techniques. However, not only do these therapies themselves often fail, they are also accompanied by discomfort and severe side effects. Unfortunately, even early complete response does not reflect overall survival since tumor recurrence is common. Therefore, TNBC is associated with increased mortality compared to other breast cancer subtypes^1^. Consequently, there is an urgent need to develop novel, low toxicity and effective therapies for TNBC. Recently, cellular therapy has drawn attention as a potential alternative therapeutic tool in regenerative medicine and for treating various chronic diseases including cancer.

Mesenchymal stromal/stem cells (MSCs), frequently isolated from bone marrow (BM), cord blood or adipose tissue, are adherent, non-hematopoietic, multipotent, fibroblast-like cells capable of differentiating into a variety of cell types including osteoblasts, chondrocytes and adipocytes. With respect to cancer progression, a number of studies have shown that MSCs exhibit a tumor-supportive role promoting tumor growth and increasing proliferation, metastasis and drug resistance during contact with tumor cells^2–4^. However, other studies have shown just the opposite, suggesting that they may have a tumor-suppressive role^5–13^.

Numerous factors, including the source tissue of the MSCs, their degree of differentiation, whether they were induced and if so by which process, the type and size of tumor being treated, the mode of MSC injection into the host animal, the treatment regimen and interactions with the host’s immune system, appear to play a role in determining whether MSCs exhibit pro-tumorigenic or anti-tumorigenic properties^4,14^. Zheng *et al*.^15^ showed that in the 4T1 BALB/c model, distal injection of mouse BM MSCs resulted in a reduction in subcutaneous (SubQ) tumor growth, angiogenesis and lung metastasis, whereas co-injection resulted in an increase in tumor growth and angiogenesis. However, they were not able to observe the same effect in nude mice, suggesting a possible role for T cells in mediating these anti-cancer effects. Yu *et al*.^16^ demonstrated that co-injection of mouse BM MSCs resulted in an increase in lung metastasis from mammary fat pad tumors when the MSCs were induced with tumor necrosis factor-alpha (TNF-α), but not when they were not induced. Similar results were reported by Du *et al*.^17^ with human BM MSCs on H460 SubQ tumors in nude mice. They showed that co-injection led to an increase in tumor growth and vascularization when the MSCs were induced with interferon-gamma (IFN-γ); whereas when the MSCs were not induced, the effect was much less. However, Yang *et al*.^18^ showed that co-injection followed up by a day 14 injection resulted in an increase in tumor growth with MSCs, but a decrease with MSCs engineered to secrete IFN-γ. Further, when MSCs are administered after tumor growth is established, the effects tend toward reduced tumor volume and mass, decreased lung metastasis, increased cell cycle arrest, increased apoptosis and inhibition of angiogenesis^19–22^.

One study on MDA-MB-231 human breast carcinoma cells, an important model representing TNBC^23^, suggested that human umbilical cord-derived MSCs attenuated cancer cell growth, inhibiting DNA synthesis in a dose-dependent manner and thereby led to tumor growth suppression^5^. This effect was attributed to stem cell secreted cytokines. Moreover, it has been shown that the secretome, which has a major part in mediating either pro- or anti-tumorigenic properties of MSCs, can be manipulated by exposing the cells to certain stimuli^19,24,25^. Several studies have suggested that pre-activation of BM MSCs with TNF-α or IFN-γ may induce anti-tumorigenic properties through expression of potentially therapeutic proteins^18,19,26^.

Human placental-derived adherent stromal cells (ASCs) are MSC-like cells which are known to possess anti-inflammatory, pro-angiogenic, cytoprotective and regenerative properties secondary to the paracrine secretion of various molecules in adaptive response to environmental stimuli^27,28^. In this study, we show for the first time that following co-induction with TNF-α and IFN-γ during the last 24 hours of their growth in culture, 3D-expanded human placental-derived ASC^29^ expression profile is changed significantly, upregulating secretion of numerous anti-proliferative, anti-angiogenic and pro-inflammatory cytokines. Conditioned medium (CM) collected from these cells inhibited the proliferation of many cancer cell lines in a 3D-spheroid screening assay. One of the most inhibited cell lines was MDA-MB-231, a known model for TNBC. We demonstrate that induced-ASCs have the capacity to halt MDA-MB-231 cell proliferation, and inhibit tumor progression and vascularization.

## Results

### Characterization of Human Placental-Derived TNF-α/IFN-γ-Induced-ASCs

**Figure 1:**
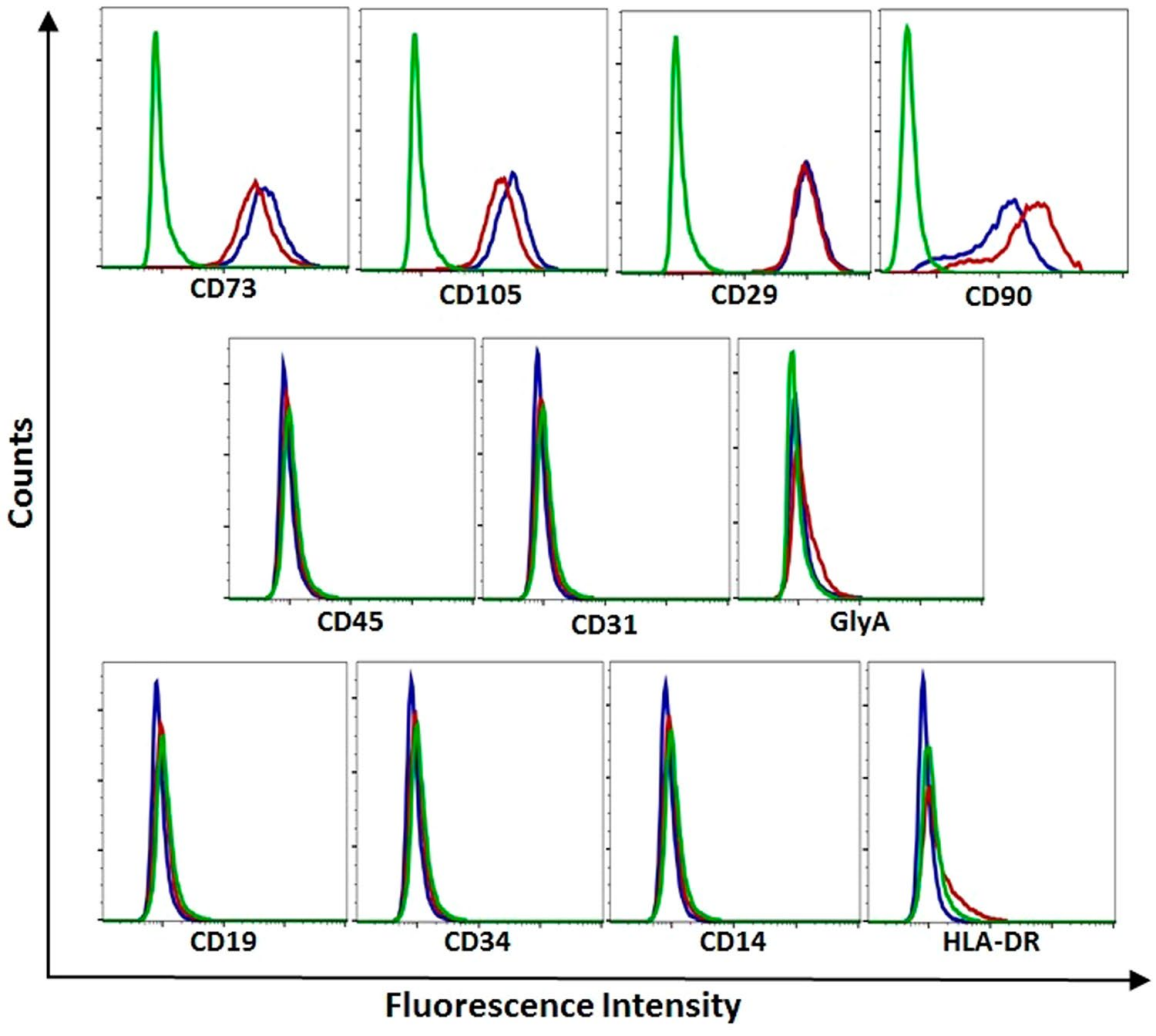
Surface Marker Expression Profiles of TNF-α/IFN-γ-Induced and Non-Induced Placental-Derived ASCs. CD marker expression in human placental-derived ASCs. Cells were stained with PE-conjugated antibodies against CD90, CD73, CD105, CD29, CD45, CD31, GlyA, CD19, CD34, CD14 and HLA-DR. CD marker expression was analyzed by flow cytometry. Isotype control IgG1 (green), TNF-α/IFN-γ-induced-ASCs (red), and non-induced-ASCs (blue).

To investigate the effect of TNF-α/IFN-γ induction on placental-derived ASC surface marker expression, induced and non-induced-ASCs were stained with phycoerythrin (PE)-conjugated monoclonal antibodies. Expression of common MSC-positive (CD90, CD73, CD105 and CD29) and MSC-negative markers (CD45, CD31, GlyA, CD19, CD34, CD14 and HLA-DR) was found to be similar between non-induced and induced human placental-derived ASCs (Fig. 1).

Secretion of cytokines implicated in either anti-tumorigenic or pro-tumorigenic processes^4,30,31^ was measured from non-induced and induced-ASCs using custom-made Luminex panels. After a thorough review of the literature (with choice limitations imposed by the availability of cytokines that can be measured using Luminex technology), 32 cytokines were analyzed. The results show (Table 1) that 12 of the cytokines (sICAM-1, CXCL9, CCL5, CXCL11, sFAS, Adiponectin, IL-31, IFN-γ, IL-18, IL-10, FLT3L and IL-1β) were significantly upregulated. An *in vitro* time course experiment showed that the upregulation in cytokine secretion was transient, with concentrations returning to non-induced levels after approximately one week in culture (Supplementary Table S1); however, this might not be the case *in vivo*, in a highly pro-inflammatory environment, since re-exposure to pro-inflammatory cytokines may maintain high secretion levels^32^.

**Table 1:** Concentration of Cytokines in the Conditioned Medium of Non-Induced and TNF-α/IFN-γ-Induced Human Placental-Derived ASCs.

Cytokine	Non-Induced ASC	Induced ASC	p value
MMP-1	24,993 ± 8267	95,800 ± 23,208	n.s.
**sICAM-1**	1379 ± 108	33,983 ± 5862	<0.01
**CXCL9**	<445 *	9138 ± 1534	<0.01
Angiopoietin-1	3392 ± 608	2821 ± 409	n.s.
**CCL5**	4 ± 1	2467 ± 431	<0.01
CXCL8	204 ± 45	2111 ± 819	n.s.
MMP-12	1371 ± 191	1358 ± 134	n.s.
IL-6	700 ± 401	1255 ± 331	n.s.
VEGF	871 ± 108	1024 ± 54	n.s.
**CXCL11**	<4*	612 ± 70	<0.001
**sFAS**	363 ± 36	567 ± 45	<0.05
OPN	892 ± 645	451 ± 176	n.s.
G-CSF	<30*	279 ± 125**	n.s.
TGF-β1	315 ± 75	302 ± 34	n.s.
**Adiponectin**	<70***	230 ± 35	<0.05
**IL-31**	<26 ± 9⁑	124 ± 21	<0.05
**IFN-γ**	<14*	42 ± 7	<0.01
TGF-β2	45 ± 19	39 ± 4	n.s.
II-17A	27 ± 6	38 ± 3	n.s.
**IL-18**	<14*	28 ± 4	<0.05
IL-12p70	<7*	15 ± 3	n.s.
**IL-10**	<2*	13 ± 2	<0.01
**FLT3L**	<10*	12 ± 1	<0.01
**IL-1β**	<2^†^	7 ± 2	<0.05

Units of concentrations are pg/mL. *Lower limit of quantification (LLOQ), **one data point < LLOQ, ***one data point > LLOQ of 53 pg/mL, ⁑one data point < LLOQ of 14 pg/mL, ^†^one data point > LLOQ of 2 pg/mL, n.s. = not statistically significant, n = 3 for non-induced-ASC, n = 6 for induced-ASC. Names of cytokines that are significantly upregulated are in bold.

### Induced-ASCs Inhibit Proliferation of Cancer Cells in 3D Assay

Gene expression (mRNA) profiling identified 460 genes that were upregulated in induced vs. non-induced-ASCs (p < 0.05). The most statistically significant pathways^33,34^ enriched in these genes include IFN-α/β and IFN-γ signaling as well as MHC class I mediated antigen processing and presentation (Supplementary Figure S1). Since interferon signaling pathways are known to modify MHC class I antigen processing and presentation^35^ and since many cancers are known to have impairment in these systems^36–39^, we hypothesized that TNF-α/IFN-γ-induced placental-derived ASCs may be useful in treating cancer. We screened 59 human cancer cell lines for their responsiveness to induced-ASC CM in a 3D-spheroid cell proliferation assay (see Methods for cell lines). We found that 48% of the cell lines had no proliferative response to the CM, whereas 44% had an anti-proliferative response (Table 2). The remaining 8% exhibited a slight proliferative effect. The 12 cell lines that had the strongest anti-proliferative response (the “responsive cell lines”) are CHL-1, NCI-H1792, RD, MDA-MB-231, 786-O, NCI-H460, 769-P, Hep-G2, SNU-449, HCC-1395, HT-29 and SW48. Interestingly, the same pathways that were overrepresented by genes upregulated in the induced-ASCs were significantly overrepresented (p < 0.05) in genes downregulated or exclusively mutated in the responsive cells (Supplementary Figure S1).

**Table 2:** Anti-Proliferative Effect of TNF-α/IFN-γ-Induced Placental-Derived ASC CM on Cancer Cell Lines in a 3D-Spheroid Cell Proliferation Assay.

Cell Line Source	Cell Lines Tested	Strong Positive Response (POC < 60%)	Marginal Positive Response (60% < POC < 80%)	No Effect (80% < POC < 120%)	Negative Response (120% < POC < 150%)
Bladder	5	0	0	4	1
Brain	4	0	1	3	0
Breast	6	2	1	0	3
Cervix	1	0	0	0	1
Colorectal	10	2	1	7	0
Head/Neck	1	0	0	1	0
Kidney	4	2	1	1	0
Liver	5	2	1	2	0
Lung	6	2	2	2	0
Muscle	1	1	0	0	0
Ovary	2	0	1	1	0
Pancreas	2	0	0	2	0
Pharynx	1	0	0	1	0
Prostate	4	0	4	0	0
Skin	4	1	1	2	0
Stomach	1	0	0	1	0
Thyroid	1	0	0	1	0
Uterus	1	0	1	0	0
TOTAL	59	12	14	28	5
		20%	24%	48%	8%

Summary of the 3D-spheroid proliferation assay. Results are listed according to the source organ of the cancer cell lines. Within each organ, “percent of cell proliferation relative to control medium” (POC) results are categorized into one of four categories: strong positive response (POC < 60%), marginal positive response (60% < POC < 80%), no effect (80% < POC < 120%) and negative response (120% < POC < 150%).

A mutation analysis was conducted to identify mutations that correlated (p < 0.05) with either the anti-proliferative or pro-proliferative response (Supplementary Dataset S1). Functional classification^40,41^ revealed that most of these genes are transcription factors and protein kinases (Supplementary Figure S2). This knowledge could be used in the future for personalizing the screening of patients’ tumors to increase likelihood of a positive therapeutic outcome.

### *In Vitro* Inhibition of Breast Cancer Cell Lines

**Figure 2:**
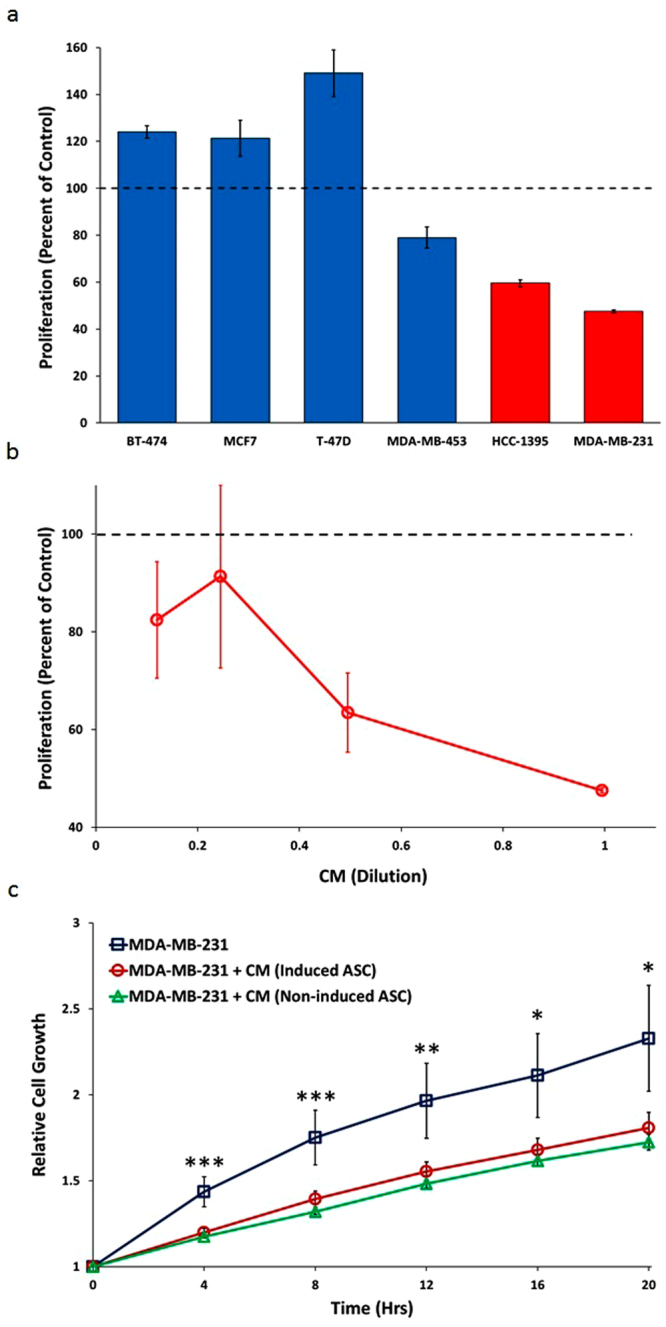
Proliferative Response of Breast Cancer Cell Lines to CM from TNF-α/IFN-γ- Induced and Non-Induced Placental-Derived ASCs. (**a**) Proliferative response of the six breast cancer cell lines to undiluted CM from TNF-α/IFN-γ-induced-ASC in the 3D-spheroid assay. Red bars represent TNBC cell lines. Error bars represent SD (n = 3). (**b**) POC response curves for MDA-MB-231 in the 3D-spheroid assay upon serial dilution. Error bars represent SD (n = 3). (**c**) Inhibition of MDA-MB-231 2D growth in real time with CM from TNF-α/IFN-γ-induced and non-induced-ASCs. Error bars represent SEM (n = 4 for MDA-MB-231 with regular growth medium, n = 11 for MDA-MB-231 + induced-ASC CM, n = 8 for MDA-MB-231 + non-induced-ASC CM). P-values are based on ANOVA: ***p < 0.01, **p < 0.01, and *p < 0.05.

Of the six breast cancer cell lines examined in the 3D-spheroid screening assay, the two cell lines derived from TNBCs, MDA-MB-231 and HCC-1395, exhibited the strongest anti-proliferative response (Fig. 2a). The POC response curve of MDA-MB-231 upon serial dilution of the CM shows that even when diluted 8 fold, inhibition was still at 18% (Fig. 2b). Since the two TNBC breast cancer cell lines responded very well to the CM, further proof of concept experiments were limited to MDA-MB-231, the most commonly studied TNBC cell line.

To examine the potential of CM derived from multiple preparations of ASCs to inhibit proliferation of MDA-MB-231, we recapitulated the 3D-spheroid cell proliferation assay by exposing MDA-MB-231 during its exponential growth phase to CM isolated not only from induced-ASCs, but also from non-induced-ASCs. The relative number of viable MDA-MB-231 cells was measured continuously for 20 hours using RealTime-GLO™. CM from both non-induced and induced-ASCs was able to significantly inhibit (p < 0.05) the growth rate of MDA-MB-231: 45% and 39% at 20 hours, respectively (Fig. 2c).

### Co-Culturing Increases CXCL11 and Angiopoietin-1 Secretion

**Figure 3:**
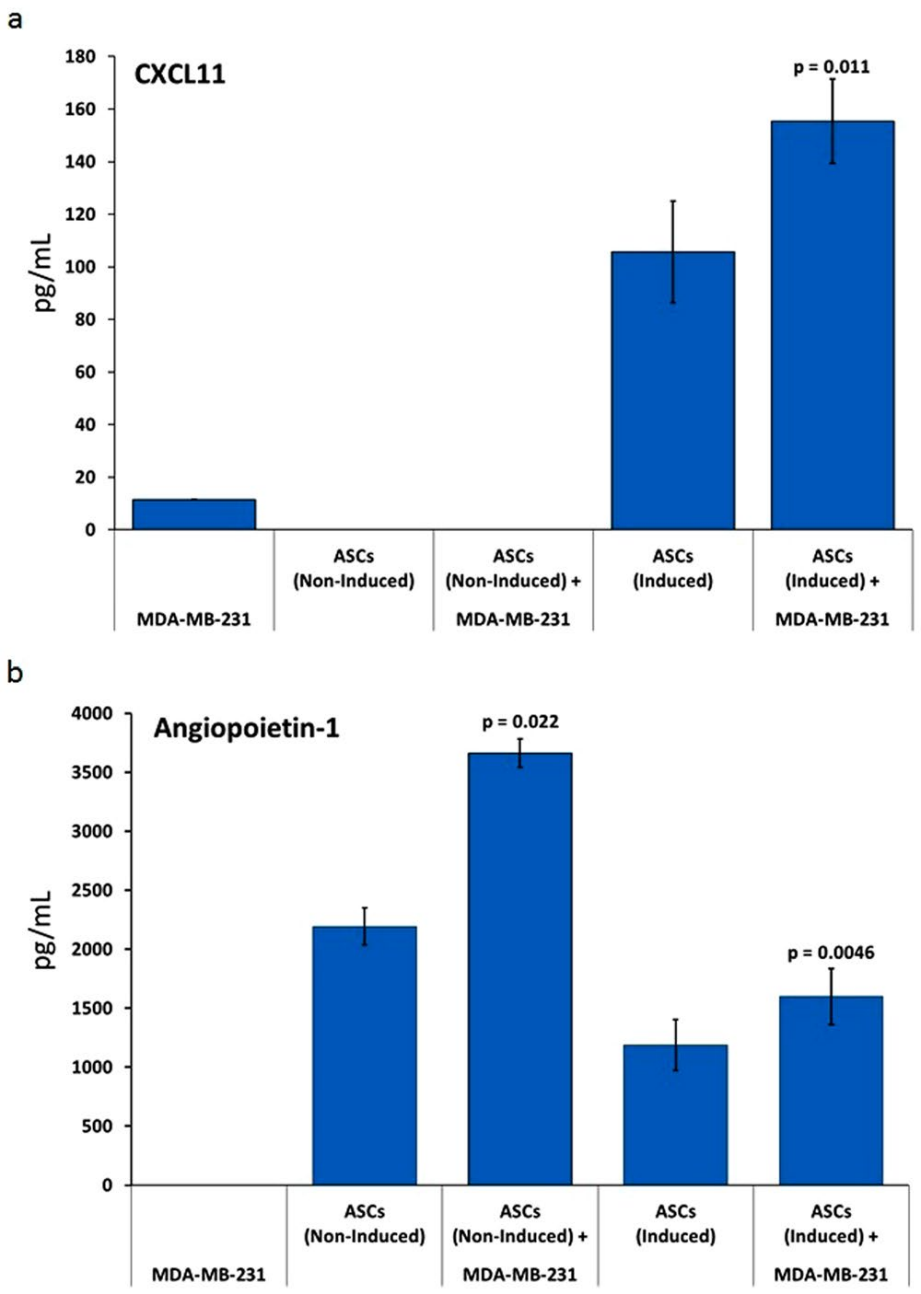
Co-Culturing of MDA-MB-231 with Human Placental-Derived ASCs Affects Cytokine Secretion Profiles. ASCs (either non-induced or induced with TNF-α/IFN-γ) were co-cultured with MDA-MB-231 and CM was collected for analysis. Error bars represent SEM (n = 3 for induced-ASCs, n = 2 for non-induced-ASCs). (**a**) CXCL11. (**b**) Angiopoietin-1. CM was collected after 48 hours. P-values are based on paired sample t-test.

Induced and non-induced-ASCs were co-cultured with MDA-MB-231 to gain insight into the mechanism of action that may be involved in cancer cell growth inhibition, analyzing the same cytokines that were analyzed above. Exposure of induced-ASCs, but not non-induced-ASCs, to MDA-MB-231 caused a significant increase (p = 0.011) in CXCL11 secretion, a potent chemotactic factor for activated T-cells, most likely derived from the induced-ASCs (Fig. 3a). Additionally, secretion of Angiopoietin-1, another ASC-derived cytokine known to inhibit angiogenesis, was upregulated significantly following co-culture of non-induced (p = 0.022) and induced-ASCs (p = 0.0046) with MDA-MB-231 (Fig. 3b). Levels of all other measured cytokines were unaffected by co-culture. These results suggest that ASCs respond to MDA-MB-231 cell secretions by upregulating the secretion of anti-tumorigenic factors. Further, the two anti-tumorigenic factors that were upregulated imply that the effect of ASCs on cancer cells is complex, most likely involving the immune and vascular systems, and that these effects cannot be recapitulated completely *in vitro*. Finally, as CXCL11 was upregulated only in the presence of TNF-α/IFN-γ-induced-ASCs, it is possible that non-induced and induced-ASCs may not have the same effects on tumors *in vivo*.

### Induced-ASCs Inhibit TNBC *in Vivo*

**Figure 4:**
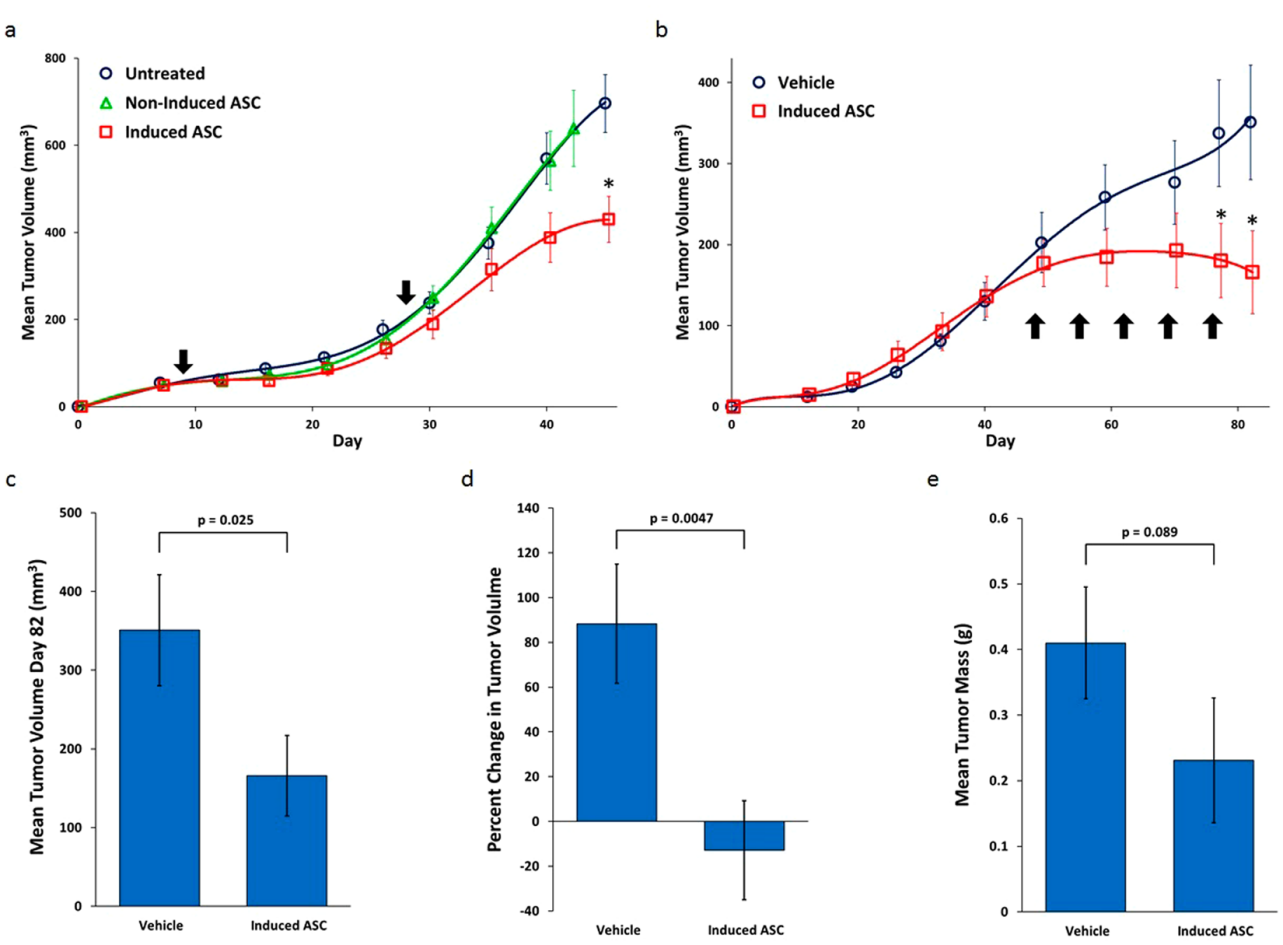
TNF-α/IFN-γ-Induced Human Placental-Derived ASCs Inhibit MDA-MB-231 Tumor Growth in Nude Mice. (**a**) Growth curves of tumor volume for MDA-MB-231 SubQ tumors in nude mice. Arrows mark days when TNF-α/IFN-γ-induced placental-derived ASCs were injected. (**b**) Growth curves of tumor volume for MDA-MB-231 mammary fat pad tumors in nude mice. The arrows mark days of the weekly IM injections of induced-ASCs. (**c**) Mean tumor volume at day 82 in the mammary fat pad model. (**d**) Percent change in mean tumor volume in the mammary fat pad model from the start of weekly IM injections of induced-ASCs. (**e**) Mean tumor mass at day 84 in the mammary fat pad model. n = 10 per group. Error bars are SEM. P-values are based on a one-tailed student’s t-test. Asterisks indicate p < 0.05.

Two *in vivo* models were studied to confirm and further characterize the biological relevance of this anti-proliferative effect. In the SubQ model, nude mice were injected in the right flank with 3 × 10^6^ MDA-MB-231 cells on day 0. One group of mice were injected intramuscularly (IM) in the right thigh with induced-ASCs on days 9 and 28. To determine the importance of TNF-α/IFN-γ induction on the anti-cancer properties of ASCs, another group of mice received ASCs that were not induced with TNF-α/IFN-γ. Mice in the control group were left untreated. Tumor progression differed dramatically between the two ASC-treated groups, especially following the second injection on day 28 (Fig. 4a). Tumors in mice that received ASCs not induced with TNF-α/IFN-γ progressed at a rate essentially identical to tumors in the untreated group, whereas tumors in mice that received ASCs induced with TNF-α/IFN-γ progressed at a slower rate. Between days 40–45, the daily growth rate of the tumors dropped by 67% in the group treated with induced-ASCs compared to the untreated group (8.4 mm^3^/day vs. 25.3 mm^3^/day), and by day 45, the mean tumor volume had decreased by 37% (430 ± 53 mm^3^ vs. 696 ± 66 mm^3^; p = 0.0029). This model suggests that treating large established tumors with induced-ASCs, as opposed to non-induced-ASCs, has a significant inhibitory effect on tumor progression.

Since treating mature tumors has potential clinical significance, we examined the extent of this effect in an orthotopic model, simulating the natural tumor microenvironment more closely. Nude mice were injected with 3 × 10^6^ MDA-MB-231 cells into the left inguinal mammary fat pad on day 0. To maximize the effect of the TNF-α/IFN-γ-induced secretion of cytokines, mice were injected IM weekly with induced-ASCs beginning on day 48. Shortly after initiation of the treatment, tumor progression was inhibited (Fig. 4b). Between days 48–59, the daily growth rate dropped 80% in the treated group compared to the vehicle group (1.2 mm^3^/day vs. 5.9 mm^3^/day), and between days 59–82, the daily growth rate decreased even further, +4.0 mm^3^/day for the vehicle group compared to −0.8 mm^3^/day for the treated group. This indicates that the tumors in the treated group had a mean negative growth rate (i.e. they exhibited shrinkage) during the final 23 days of measurements.

A 53% decrease in mean tumor volume (Fig. 4c) was observed by the end of the study (p = 0.025). However, since treatment with induced-ASCs did not begin until day 48, to obtain a more relevant measure of the effect of induced-ASCs on tumor progression, we calculated the percent change in mean tumor volume from days 48-82 (Fig. 4d). During this period, the mean tumor volume of the vehicle group increased by 88%, whereas the mean tumor volume in the treated group decreased by 13% (p = 0.0047). Finally, tumor mass was determined on the day of sacrifice to be 44% lower in the treated group than in the vehicle group (Fig. 4e), 0.23 ± 0.1 g vs. 0.41 ± 0.09 g (p = 0.089). Notably, 30% of the mice in the ASC treated group experienced complete remission by day 84 compared to 0% in the vehicle treated group.

### Induced-ASCs Decrease MDA-MB-231 Cell Proliferation and Tumor Vascularization

**Figure 5:**
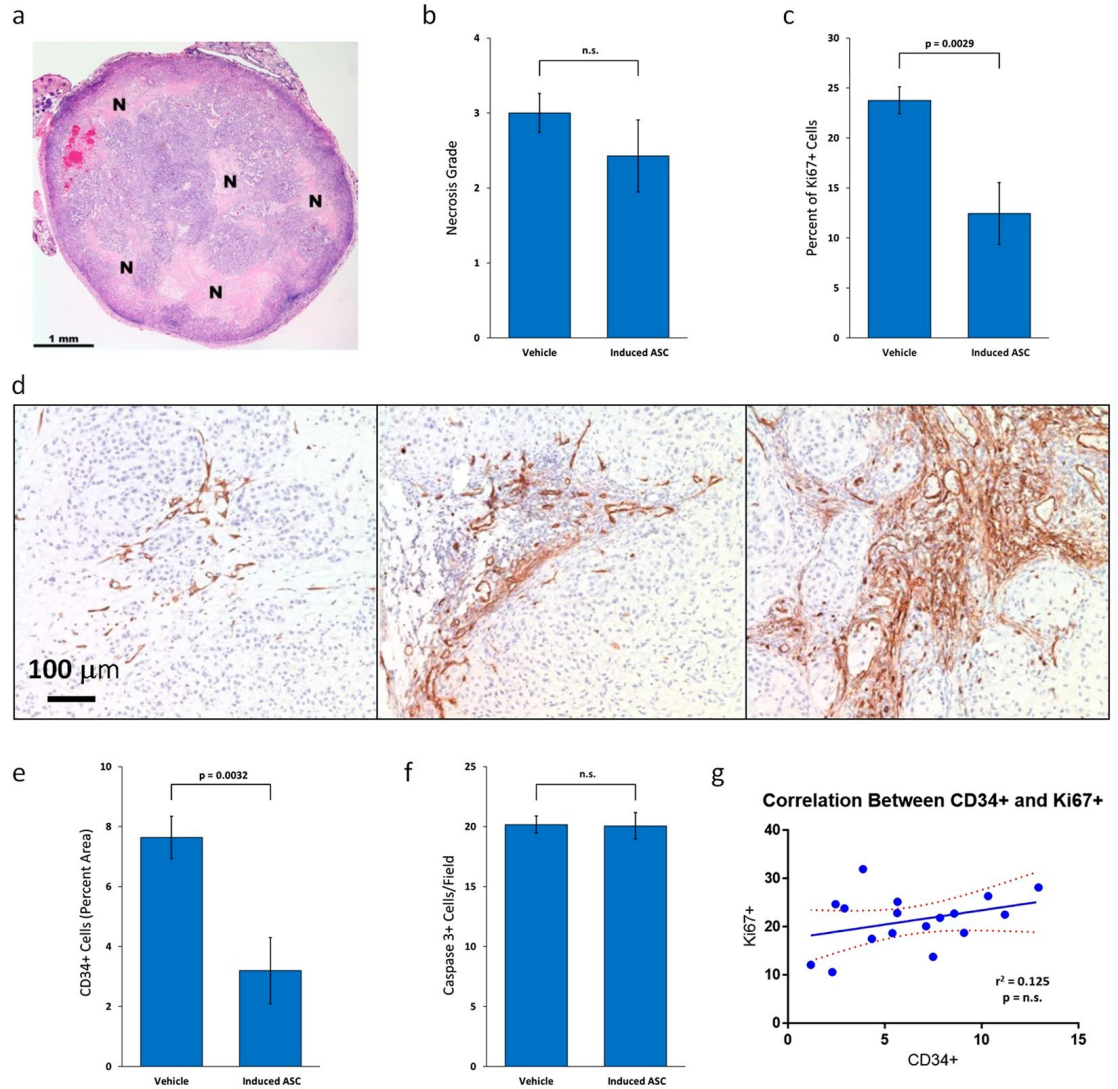
TNF-α/IFN-γ-Induced Human Placental-Derived ASCs Inhibit Tumor Cell Proliferation and Vascularization in MDA-MB-231 Tumors in Nude Mice. (**a**) Representative section of H&E-stained MDA-MB-231 mammary fat pad tumor with grade 2 necrosis from mouse treated with TNF-α/IFN-γ-induced placental-derived ASCs. “N” marks areas of necrosis. (**b**) Mean necrosis grade in MDA-MB-231 mammary fat pad tumors from nude mice treated with either induced-ASCs or vehicle. (**c**) Mean percentage of proliferating cells in MDA-MB-231 mammary fat pad tumors from nude mice treated with either induced-ASCs or vehicle. (**d**) Representative sections of CD34 immunostained MDA-MB-231 mammary fat pad tumors showing different levels of vascularization all from different areas of tumors from mice treated with induced-ASCs. (**e**) Mean area occupied by blood vessels in MDA-MB-231 mammary fat pad tumors from nude mice treated with either induced-ASCs or vehicle. (**f**) Mean active caspase 3-expressing cells per field in MDA-MB-231 mammary fat pad tumors from nude mice treated with either induced-ASCs or vehicle. (**g**) Correlation between percent CD34+ stained area and percent Ki67+ cells in MDA-MB-231 mammary fat pad tumors in nude mice. n = 10. Error bars are SEM. P-values are based on a one-tailed student’s t-test.

H&E staining revealed tumors composed of cells with epithelioid morphology, abundant cytoplasm and pleomorphic nuclei with large nucleoli, consistent with breast carcinoma cells. No apparent morphological differences were observed between tumors of the two groups. Most of the tumors showed prominent necrosis (Fig. 5a); however, there was no difference in mean necrosis grade between the treated and non-treated groups (Fig. 5b). To measure the levels of cell proliferation in the tumors, sections were stained for Ki67. Figure 5c shows that 23.8 ± 1.4% of the tumor cells stained for Ki67 in the vehicle group, compared to only 12.5 ± 3.1% in the treated group, representing a 48% reduction in proliferating cells (p = 0.0029). To determine if induced-ASCs inhibited cancer progression by reducing the level of vascularization of the tumors, sections were immunostained with anti-CD34 (Fig. 5d). Figure 5e shows that the area occupied by CD34+ cells decreased by 58% in tumors from the treatment group compared to the vehicle group (3.2 ± 1.0% to 7.6 ± 1.0%, respectively; p = 0.0032). There was no correlation (r^2^ = 0.018, p = 0.61) between the area occupied by CD34+ cells and tumor mass, suggesting that the observed decrease in tumor mass is not solely due to the decrease in vascularization. Tumor sections were also immunostained for active caspase 3, and the number of apoptotic cells was counted. Figure 5f shows that there was no difference in the number of apoptotic cells between the two groups. Finally, the results show no correlation between percentage of Ki67+ cells and CD34+ area in each tumor, suggesting that the induced-ASCs inhibit tumor growth by at least two separate mechanisms (Fig. 5g).

### Lymph Node and Lung Micro-Metastases

**Figure 6:**
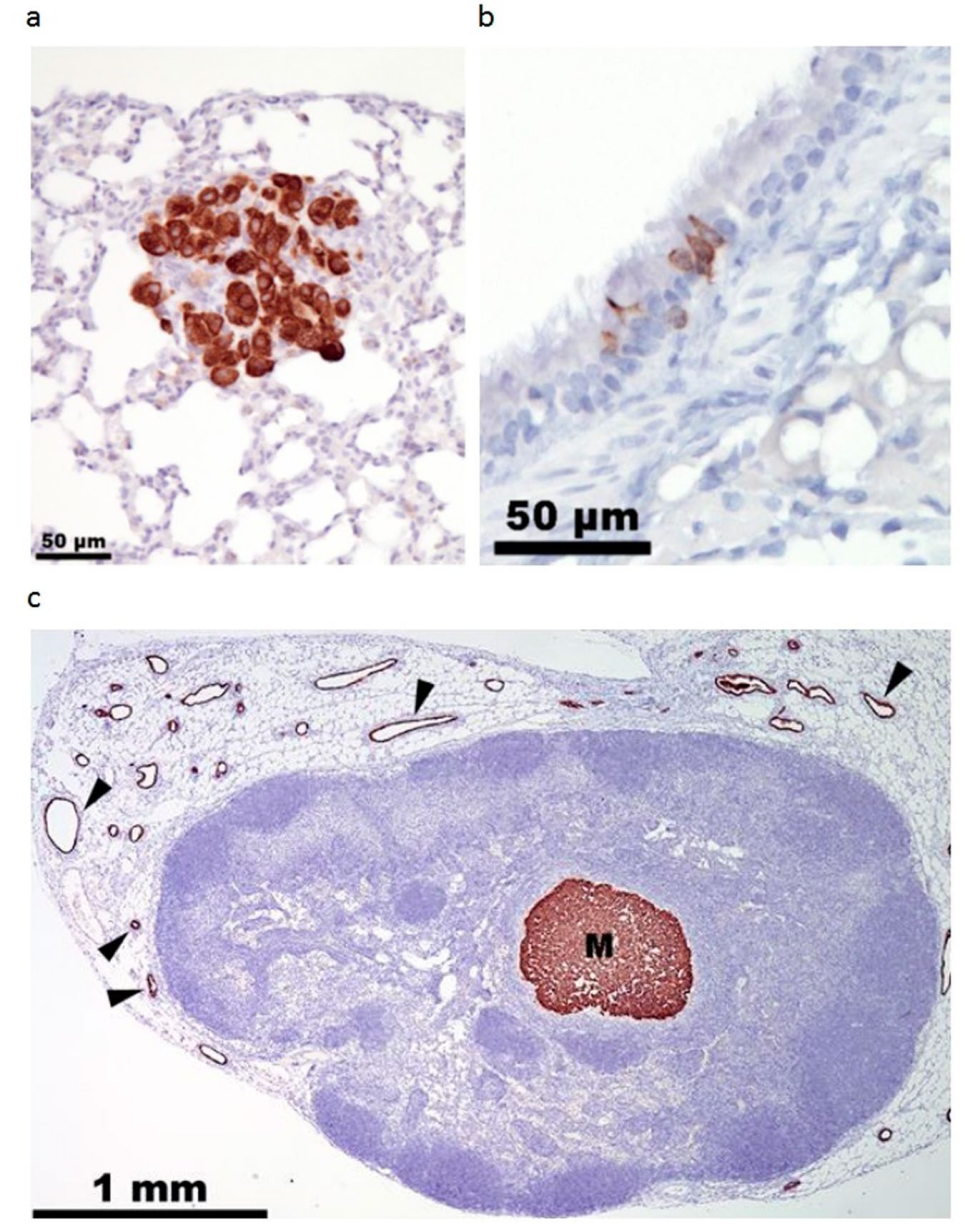
Lymph Node and Lung Micro-Metastases. (**a**) Representative MDA-MB-231 metastatic cells in lung section immunostained for CK18. (**b**) A group of normal respiratory epithelial cells showing weak immunostaining for CD18. (**c**) Representative metastasis (labeled “M”) in lymph node section immunostained for CD18. Black wedges mark endothelial cells in mammary gland ducts surrounding lymph nodes.

Since lungs are a primary metastatic organ for breast cancer, lung samples from each mouse were stained for cytokeratin 18 (CK18) to detect metastasized tumor cells (Fig. 6a). Microscopic analysis revealed occasional staining of normal respiratory epithelial cells lining bronchi and bronchioles in all samples and in single pneumocytes (Fig. 6b). Metastatic tumor cells differed from these cells by their larger size and stronger CK18 immunostaining. Treatment with TNF-α/IFN-γ induced-ASCs eliminated lung metastases (Table 3).

**Table 3:** Distribution of Lymph Node and Lung Metastases in Vehicle and TNF-α/IFN-γ-Induced Placental-Derived ASC Treated Groups.

Vehicle			Induced-ASCs		
Mouse	LN Mets	Lung Mets	Mouse	LN Mets	Lung Mets
V1	lumbar	—	T1	—	—
V2	—	—	T2	—	—
V3	axillary, lumbar	√	T3	—	—
V4	axillary, lumbar	√	T4	—	—
V5	—	—	T5	lumbar	—
V6	—	—	T6	axillary	—
V7	—	—	T7	lumbar	—
V8	—	—	T8	lumbar	—
V9	axillary, lumbar	—	T9	lumbar	—
V10	—	—	T10	—	—

Three pairs of lymph nodes (axillary, inguinal and lumbar) were stained with anti-CK18 (see Fig. 6c for an example). Results show that none of the mice had micro-metastases in their inguinal lymph nodes: 40% of the mice from each group had at least one micro-metastasis in their lumbar lymph nodes, and 30% of the mice from the vehicle group had micro-metastases in their axillary lymph nodes, compared with 10% in the treated group (Table 3). These data suggest that induced-ASCs did not reduce metastasis from the mammary fat pad to the lumbar lymph nodes, but did reduce metastasis to the axillary lymph nodes.

### Induced-ASCs Reverse the Increase of Pro-Tumorigenic Murine Cytokines in Serum

**Figure 7:**
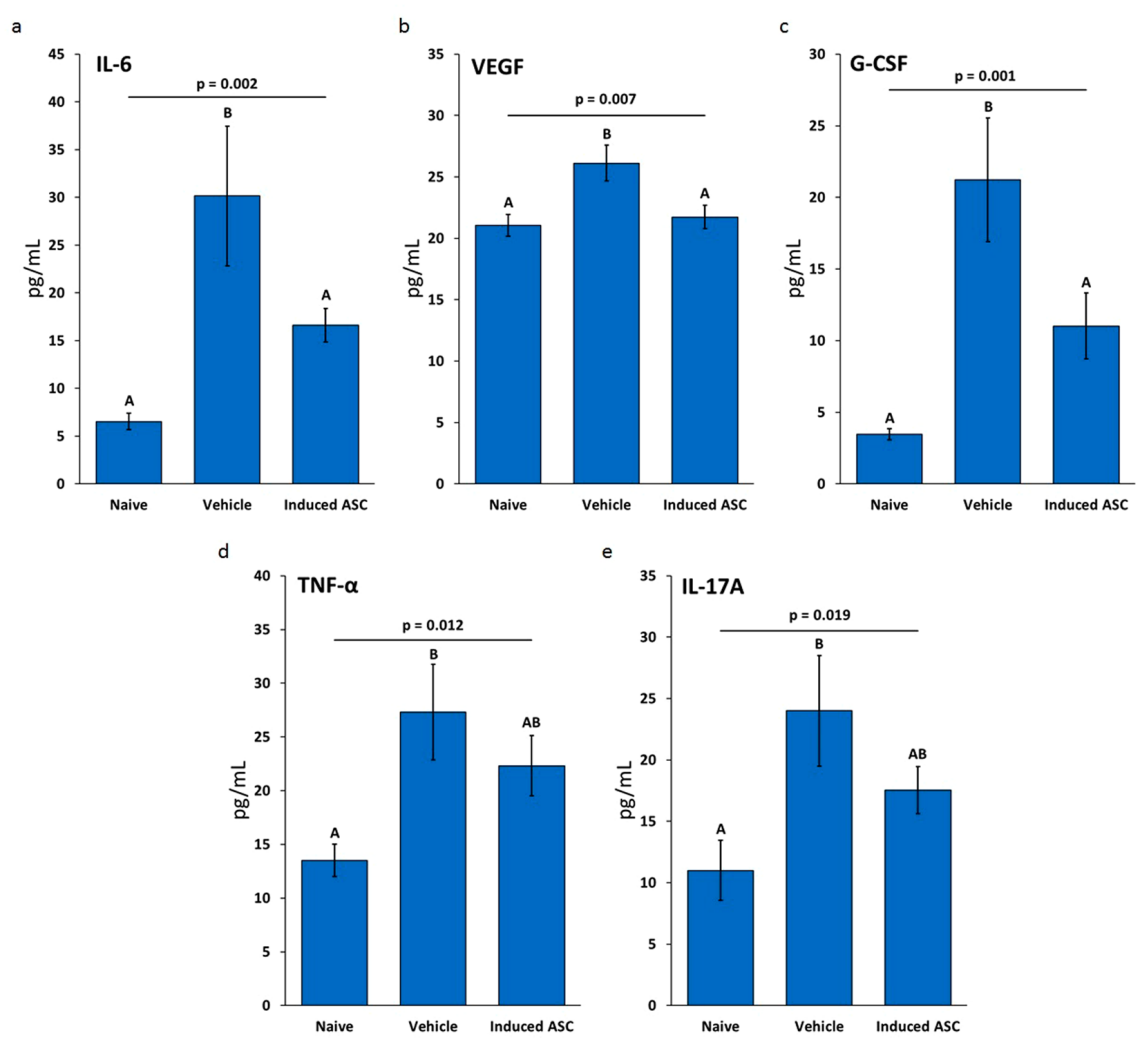
TNF-α/IFN-γ-Induced Placental-Derived ASCs Reverse the Increased Concentration of Pro-Tumorigenic Murine Cytokines in the Serum of Nude Mice Bearing MDA-MB-231 Tumors. Concentration of murine cytokines in the serum of nude mice. (**a**) IL-6. (**b**) VEGF. (**c**) G-CSF. (**d**) TNF-α. (**e**) II-17A. Data are based on n = 10 with outliers removed prior to performing statistical analysis. One way ANOVA was performed with the Duncan *post hoc* test. “A” and “B” indicate groups resulting from the *post hoc* test (α < 0.05). Error bars are SEM.

Murine cytokine protein expression in serum samples isolated from blood taken at the end of the study (day 84) from vehicle-treated and induced-ASC-treated mice was compared to expression in serum from age-matched naïve mice (n = 10 in each group). Out of 24 cytokines examined, 13 were expressed at detectable levels. A one-way analysis of variance (ANOVA) demonstrated an effect on five of the cytokines: IL-6 (p = 0.002), VEGF (p = 0.007), G-CSF (p = 0.001), TNF-α (p = 0.012) and II-17A (p = 0.019). A Duncan *post hoc* test showed that for IL-6, VEGF and G-CSF, the vehicle-treated group expressed significantly higher levels compared to both naïve and ASC-treated mice. Although TNF-α and II-17A expression was significantly upregulated in untreated tumor-bearing mice compared to naïve mice, their expression in the sera of the induced-ASC treated group was reduced relative to the vehicle group but not back to naïve levels (Fig. 7).

## Discussion

Currently, there is no targeted therapy for TNBC. The only options are a combination of chemotherapy, surgery and radiation techniques. Several pre-clinical trials have explored the potential of cellular therapy, with varying results^5,23^. Here we report for the first time, that exposure of human placental-derived ASCs to TNF-α/IFN-γ in the last 24 hours of their culture in a 3D bioreactor growth system induces significant changes in their secretion profile and *in vivo* potency. The resulting cells exhibit an anti-proliferative effect on a wide range of cancer types in 3D-spheroid cultures. This property has been validated for TNBC xenograft tumors in mice. The induced-ASCs also exert an anti-angiogenic and possibly an anti-metastatic effect on primary tumors *in vivo*.

TNF-α/IFN-γ-induced placental-derived ASCs express classical MSC CD markers, not differing from non-induced placental ASCs in this regard (Fig. 1). However, gene expression profiling revealed major differences in hundreds of genes, many of which belong to pathways involved in immune responses (Supplementary Figure S1). Some of these genes were also upregulated at the protein level (Table 1), with many of the cytokines having known anti-tumorigenic effects, i.e. CXCL9^42^ and IL-31^43^ are anti-angiogenic, sICAM-1^44^ is known to inhibit epithelial-mesenchymal transition (EMT), sFAS^45^ inhibits metastasis, CXCL9^46–48^, CXCL11^47,49,50^, IFN-γ^51^, FLT3L^52^ and IL-31^53^ are able to induce recruitment of pro-inflammatory, anti-tumorigenic immune cells, IL-10^54^ and sFAS^55^ affect tumor immune rejection, and Adiponectin^56^ is anti-proliferative. On the other hand, it is important to note that some of these cytokines may also have pro-tumorigenic effects in some situations. For example, sICAM-1, although it functions as an EMT inhibitor, is also known to increase angiogenesis and tumor metastatic potential^57^. CCL5, although it is well known for its pro-tumorigenic role^31,58,59^, has recently been promoted as a natural adjuvant for enhancing anti-tumor immune responses^60^. Finally, the concentration of a given cytokine as measured *in vitro* may not reflect its concentration *in vivo* especially after multiple injections of ASCs and exposure to a totally different microenvironment. It is thus possible that even cytokines with low concentrations *in vitro* may have elevated concentrations *in vivo* and contribute to the anti-tumorigenic effect.

To examine the anti-proliferative potential of the induced-ASCs, we screened many types of cancer cell lines in a 3D-spheroid cell proliferation assay using CM derived from induced-ASCs. A 3D assay was chosen because a growing body of evidence suggests that 3D culture systems more accurately recapitulate the microenvironment where cells reside in tissues, including the fact that cells in a 3D environment differ morphologically and physiologically from cells in a 2D environment^61^. Ninety-two percent of the cell lines tested either were inhibited or were unaffected by induced-ASCs (Table 2). Thus, even though certain MSC preparations have been shown by others to stimulate cancer cell growth, our 3D-expanded human placental-derived induced-ASCs have broad anti-proliferative properties. Indeed, 44% of the cell lines tested exhibited an anti-proliferative response to the induced-ASC CM.

Analysis of known somatic mutations in the cancer cell lines identified mutations which are highly correlated to either the anti-proliferative or pro-proliferative response (Supplementary Dataset S1 and Supplementary Figure S2). This knowledge could be used in the future for personalized screening of patients’ tumors to significantly increase the likelihood of a positive therapeutic outcome with induced-ASCs treatment. We bring two examples to illustrate. Mutations in PTEN (phosphatase and tensin homolog), one of the most common tumor suppressor genes known to be mutated in cancers, are positively correlated with the anti-proliferative response (log effect = −0.20; p = 3 × 10^−7^), indicating that women with these mutations may respond favorably to treatment with the induced-ASCs. On the other hand, mutations in LATS1 (large tumor suppressor kinase 1), a gene which encodes a putative serine/threonine kinase that localizes to the mitotic apparatus and is associated with the Hippo signaling pathway, are positively correlated with the pro-proliferative response (log effect = 0.17; p = 0.0026), indicating that women with these mutations may not respond favorably to treatment.

Since the two breast cancer cell lines that were most responsive to the CM were both TNBC lines (Fig. 2a) and because there is a serious unmet need in this field of oncology, we focused on MDA-MB-231 as the model cell line. *In vitro* studies confirmed that CM from induced-ASCs was able to inhibit the proliferation of MDA-MB-231 cells also in 2D cell cultures. This was important because it enabled the testing of multiple preparations of ASCs including non-induced-ASCs, and it validated the culture system for other *in vitro* studies. Surprisingly, CM from the non-induced-ASCs had a similar inhibitory effect as induced-ASCs (Fig. 2c). However, secretion analysis of induced-ASCs when co-cultured with MDA-MB-231 cells revealed that there may be differences in response between non-induced and induced-ASCs. Whereas secretion of Angiopoietin-1, known to inhibit angiogenesis, was upregulated in response to MDA-MB-231 cells by both non-induced and induced-ASCs (Fig. 3b), only induced-ASCs secreted and further upregulated the secretion of CXCL11, a potent chemotactic factor for activated T-cells (Fig. 3a). Taken together, these data indicate that potential additional pathways which cannot be recapitulated *in vitro* may be operational *in vivo* and have effects through immune cells and blood vessels^50,62^. As a result, we utilized ectopic and orthotopic mouse models to test the efficacy of ASCs on MDA-MB-231 tumors *in vivo*.

A SubQ nude mouse model demonstrated that the anti-proliferative effect observed *in vitro* can be observed *in vivo*, and that although non-induced-ASCs were able to inhibit the growth of MDA-MB-231 in 2D cell culture, they were ineffective at inhibiting the growth of MDA-MB-231 tumors *in vivo* (Fig. 4a). However, and perhaps more importantly, in light of the controversy surrounding the clinical use of MSCs due to their potential pro-tumorigenic effect, these non-induced-ASCs did not stimulate growth of the tumors. Furthermore, the inhibitory effect of induced-ASCs on tumor progression was observed when ASCs were injected into mice that bore established tumors, emphasizing the clinical potential of the IM treatment. As we and others have shown, IM administered MSCs remain restricted to the muscle tissue where they persist for a limited period and induce a systemic effect. Therefore, we consider this to be a safe mode of administration^63,64^.

Many studies suggests that the MSC injection protocol plays a critical role in determining whether MSCs exert a positive or negative effect on tumor progression^15–18^. In NOD/SCID and nude mice, MSCs administered after MDA-MB-231 tumors were established, led to reduced tumor volume and mass, decreased lung metastasis, increased cell cycle arrest, increased apoptosis and inhibition of angiogenesis^19–22^. Therefore, the second model was designed with several key features to maximize the anti-tumorigenic potential of the induced-ASCs. Tumors would be implanted in the mammary fat pad to simulate the tumor microenvironment more accurately, treatment would commence once tumors were established, and since the duration of the observed upregulation of secreted cytokines *in vivo* was unknown ASCs would be administered on a weekly basis.

The second *in vivo* study yielded much better results than the initial SubQ model in that tumor growth was not only retarded, but rather halted altogether (Fig. 6b and d). Further, 30% of the mice in the treated group experienced complete tumor remission compared to 0% in the vehicle group. Tumor histopathology showed that induced-ASCs affected tumor growth by inhibiting cell proliferation and tumor vascularization (Fig. 7c and e). The latter effect may be mediated in part by the >20 fold increase in CXCL9 secretion and the >5 fold increase in IL-31 secretion from the induced-ASCs that was observed *in vitro*^42,43^. Other cytokines that are known to inhibit angiogenesis include MMP-12^65,66^ and Angiopoietin-1^62,67^. These were also secreted at high concentration by induced-ASCs (Table 1), with Angiopoietin-1 secretion being enhanced further by co-culturing (Fig. 3b). However, the anti-tumorigenicity of induced-ASCs did not appear to be mediated by tumor necrosis or apoptosis (Fig. 7b and f). The same conclusion was supported by *in vitro* data, in which MDA-MB-231 growth inhibition seemed to slow down tumor cell proliferation rather than induce cell death (Fig. 2c). Additionally, the data suggests that induced-ASCs may reduce tumor metastasis to the lungs through the axillary lymph nodes (Table 3), an effect potentially mediated by the enhanced secretion of sFAS by induced-ASCs (Table 1). The fact that the induced-ASCs have a significant anti-tumor effect on larger tumors rather than on smaller tumors suggests that they may be applicable for treating large inoperable tumors. Some of the cytokines that were upregulated by induction of ASCs are reported to have anti-tumorigenic properties through activation and chemotaxis of cytotoxic T cells, i.e. CXCL11, IL-31, IFN-γ and FLT3L. Since both *in vivo* experiments were performed in nude mice (to avoid rejection of the human MDA-MB-231 cells), the immunomodulatory effect of induced-ASCs is probably underestimated in these models. Future studies should be conducted in immunocompetent or humanized mice to enable a more complete assessment of the immunomodulatory arm of this potential cell therapy.

When examining murine cytokines in mice sera collected at the day of sacrifice, we found that IL-6, VEGF, G-CSF, TNF-α and II-17A, all known to be overexpressed in tumors and sera of cancer patients and which contribute to tumor proliferation and invasion^21^, were all significantly upregulated in the presence of tumors. Mean concentrations of IL-6, G-CSF and VEGF were restored to naïve levels by induced-ASCs, and a decrease in mean concentrations of II-17A and TNF-α was also observed, although not to naïve levels (Fig. 6). Interestingly, although induced-ASCs secrete considerable amounts of the potentially tumorigenic cytokines IL-6 and VEGF, when administered *in vivo* murine concentrations of IL-6 and VEGF are significantly lowered, showing that *in vivo* effects of ASCs may not be easily predicted from *in vitro* data. The combined effect of the cells *in vivo* is complex and multifaceted, made even more complex by the fact that these cells actively respond to multiple stimuli from their surroundings.

In conclusion, the secretome of human placental-derived ASCs may be transiently modified by adjusting their manufacturing process. Such modifications result in a dramatic effect on *in vivo* functionality. Second, the anti-tumorigenic effects of the TNF-α/IFN-γ-induced-ASCs on TNBC *in vivo* are dependent on tumor size, suggesting the importance of crosstalk between induced-ASCs and tumor cells. Third, since these studies were conducted using multiple batches of ASCs isolated from different placentas, the 3D cell expansion and induction protocol does not rely on a single cell source for stability, consistency and reliability of results. Finally, many of the cytokines secreted by the induced-ASCs are known to interact with cells of the immune system, especially T cells (which are lacking in nude mice), suggesting that the full potential of these human placental-derived ASCs on mitigating TNBC cancers may not yet have been fully realized.

## Methods

### Production and Culturing of Adherent Stromal Cells (ASCs)

Adherent stromal cells (ASC) were produced and supplied by Pluristem Ltd. (Haifa, Israel). ASCs are produced from full-term placentas following elective Caesarean section. ASC production is composed of two major steps of isolation and culturing of the adherent stromal cells in tissue culture flasks and a 3D growth phase on non-woven fiber made carriers in controlled bioreactors for further expansion as previously described^29^.

### Induction of ASCs

To induce ASCs, TNF-α and IFN-γ (each at 10 ng/mL, ≥200 units/mL), both purchased from PeproTech, were added for the last 24 hours of 3D culture. During growth in the bioreactor, medium is constantly refreshed using perfusion based on the cells’ glucose consumption. Hence TNF-α and IFN-γ are also refreshed during the 24 hours of induction.

### Preparation of Conditioned Medium (CM)

After cryopreservation, 5 × 10^5^ ASCs were seeded in 6-well plates in DMEM low glucose supplemented with 2 mM L-glutamine, 10% FBS and 50 µg/ml gentamycin. After 24 hours, the medium was aspirated, cells were washed, and DMEM high glucose (supplemented with L-glutamine and gentamycin but without FBS) was added. After 24 hour incubation, the medium was collected, centrifuged, aliquoted and kept in −80 °C until use.

### ASC Membrane Markers Phenotype Assay

Cells were stained with phycoerythrin (PE) conjugated monoclonal antibodies and appropriate isotype controls for the characteristic MSC-positive markers (CD90, CD73, CD29 and CD105) and MSC-negative markers (CD34, CD45, CD19, CD14, CD31, GlyA and HLA-DR) all supplied by BD Biosciences. All the membrane marker tests were performed using the FC500 Flow cytometry system (Beckman Coulter, Fullerton, CA, USA) with CXP analysis software.

### *In Vitro* Secretion Profile of ASCs

CM was collected as described above from three preparations of non-induced-ASCs and six preparations of induced-ASCs. The three preparations used to prepare the non-induced-ASCs were from different placentas, and they were also used to prepare four out of the six induced-ASC preparations. The other two induced-ASC preparations were from another placenta. CM was tested for expression of Adiponectin, Angiopoietin-1, FMS-like tyrosine kinase 3 ligand (FLT3L), Granulocyte-colony stimulating factor (G-CSF), Interferon beta (IFN-β), Interferon gamma (IFN-γ), Interleukin 1 beta (IL-1β), Interleukin-6 (IL-6), Chemokine (C-X-C motif) ligand 8 (CXCL8), Interleukin-10 (IL-10), Interleukin-12 heterodimer (IL-12p70), Interleukin-15 (IL-15), Interleukin-17A (II-17A), Interleukin-18 (IL-18), Interleukin-21 (IL-21), Interleukin-31 (IL-31), C-X-C motif chemokine 11 (CXCL11), Chemokine (C-X-C motif) ligand 9 (CXCL9), Macrophage inflammatory protein 1 alpha (MIP-1α), Matrix metalloproteinase-1 (MMP-1), Matrix metallopeptidase-12 (MMP-12), Osteopontin (OPN), Chemokine (C-C motif) ligand 5 (CCL5), Soluble FAS receptor (sFAS), Soluble intercellular adhesion molecule-1 (sICAM-1), Transforming growth factor beta 1, 2 and 3 (TGF-β1/2/3), Tumor necrosis factor beta (TNF-β), TNF-related apoptosis-inducing ligand (TRAIL) and Vascular endothelial growth factor (VEGF) using Luminex-based custom-made assays from either R&D systems or eBioscience as per manufacturer’s protocol. Statistical analysis was performed using independent sample t-test.

### *In Vitro* 3D-Spheroid Cell Proliferation

The *in vitro* 3D-spheroid cell proliferation assay was conducted at Bioensis (Bellevue, WA, USA). The human cancer cell lines 22Rv1 (ATCC CRL-2505), 647-V (ATCC ACC-414), 769-P (ATCC CRL-1933), 786-O (ATCC CRL-1932), A-498 (ATCC HTB-44), A-549 (ATCC CCL-185), ACHN (ATCC CRL-1611), AGS (ATCC CRL-1739), AsPC-1 (ATCC CRL-1682), BT-474 (ATCC HTB-20), C32 (ATCC CRL-1585), C3A (ATCC CRL-10741), Cal 27 (ATCC CRL-2095), CAL-62 (ATCC ACC 448), Calu-6 (ATCC HTB-56), CHL-1 (ATCC CRL-9446), Colo-205 (ATCC CCL-222), Colo-320-HSR (ATCC CCL-220.1), COLO-829 (ATCC CRL-1974), DBTRG-05MG (ATCC CRL-2020), DLD-1 (ATCC CCL-221), DU-145 (ATCC HTB-81), ES-2 (ATCC CRL-1978), FaDu (ATCC HTB-43), HCC-1395 (ATCC CRL-2324), HCT-116 (ATCC CCL-247), HCT-15 (ATCC CCL-225), Hela (ATCC CCL-2), Hep 3B2.1-7 (ATCC HB-8064), Hep-G2 (ATCC HB-8065), HT-1376 (ATCC CRL-1472), HT-29 (ATCC HTB-38), Huh7 (ATCC Huh7), J82 (ATCC HTB-1), LNCaP clone FGC (ATCC CRL-1740), LS-174 T (ATCC CL-188), MCF7 (ATCC HTB-22), MDA-MB-231 (ATCC HTB-26), MDA-MB-453 (ATCC HTB-131), MES-SA (ATCC CRL-1976), Mia PaCa-2 (ATCC CRL-1420), NCI-H1792 (ATCC CRL-5859), NCI-H23 (ATCC CRL-5800), NCI-H358 (ATCC CRL-5807), NCI-H460 (ATCC HTB-177), PC-3 (ATCC CRL-1435), RD (ATCC CCL-136), SK-MEL-3 (ATCC HTB-69), SK-N-AS (ATCC CRL-2137), SK-OV-3 (ATCC HTB-77), SNU-449 (ATCC CRL-2234), SW1088 (ATCC HTB-12), SW48 (ATCC CCL-231), SW480 (ATCC CCL-228), SW620 (ATCC CCL-227), T24 (ATCC HTB-4), T-47D (ATCC HTB-133), U-87 MG (ATCC HTB-14), UM-UC-3 (ATCC CRL-1749) were grown in RPMI+10% FBS, 2 mM L-alanyl-L-glutamine, and 1 mM sodium pyruvate. Cell lines were seeded in the above medium but with 5% FBS and 1% penicillin/streptomycin to form spheroids, in multi-well 3D plates (Elplasia™ plates) pre-coated with pHEMA. ASC CM (prepared as described above but in RPMI medium instead of DMEM high glucose) was supplemented with 5% FBS and added either neat or diluted 1:2, 1:4, or 1:8 to the spheroids 24 hours post seeding. Cells were incubated for seven days with one medium exchange at day 3. RPMI with 5% FBS and staurosporine were included as controls for every cell line. Cells were lysed and analyzed with CellTiter-Glo® following the manufacturer’s protocol to determine cell viability.

### Global Gene Expression Analysis in ASCs

A whole genome expression assay was performed on TNF-α/IFN-γ induced and non-induced-ASCs. ASCs were thawed into DMEM medium (containing 10% FBS) and RNA was extracted immediately using RNeasy Mini Kit (Qiagen) and quantified by a NanoDrop spectrophotometer. Quality measurements for total RNA were performed using TapeStation (Agilent). The RINe values of all samples was >9.5. The RNA was amplified into biotinylated cRNA by *in vitro* transcription using the TargetAmp Nano labeling kit for Illumina BeadChips (Epicentre) with 100 ng of total RNA as input material. Biotinylated cRNAs were purified, fragmented, and subsequently hybridized to an Illumina HumanHT-12 v4 Expression BeadChip according to the Direct Hybridization assay (Illumina Inc.). The hybridized chip was stained with streptavidin-Cy3 (Amersham™) and scanned with an Illumina HiScan.

The raw gene expression data was exported from GenomeStudio and imported into JMP Genomics V7 software (SAS Institute Inc., Cary, NC). Quality control and analysis in JMP Genomics was done on log_2_ transformed data, after filtering for non-expressed genes (detection p-value < 0.01), and for low variance transcripts across samples (variance <5%). Since data distribution showed that expression between samples was uniform, data was not normalized. The Comparative Marker Selection module^68^ in GenePattern^69^ was utilized to detect differentially expressed genes. The t-test was used to assign rank to each gene. Statistical significance was determined by a gene specific p-value based on permutation testing <0.05.

### Global Gene Expression and Somatic Mutation Analyses in Cancer Cell Lines

Gene expression was assessed using the publically available Broad-Novartis Cancer Cell Line Encyclopedia^70^ Affymetrix U133 + 2 arrays. Raw Affymetrix CEL files from the Affymetrix U133 + 2 arrays were converted to a single value for each probe set using Robust Multi-array Average (RMA) and normalized using quantile normalization. A redefined custom CDF file from the package HGU133Plus2_Hs_ENTREZG_15.0.0 from Brainarray^71^ was used for the summarization. Cell lines that were strongly inhibited, i.e. that had a “percent of cell proliferation relative to control medium” (POC) proliferation ≤60%, were compared to cell lines that were not inhibited (i.e. POC ≥ 79%). Marginally responsive cell lines defined as having 60% <POC <79% were excluded from both classes. CHL-1 and RD were excluded from this analysis due to statistical limitations. Five comparisons were performed, each being restricted to cell lines within the same organ. Differentially expressed genes were determined as above.

Using the publically available COSMIC Cancer Cell Lines Project somatic mutations database^72^, known somatic mutations from full exome sequencing (e.g. additions, deletions, substitutions, frameshifts, splice sites) were analyzed in the cancer cell lines (except for Hep-G2, AGS, DLD1, LS-174 T and SW480 which were not included in the database). CDS silent mutations and intronic mutations were not considered mutations for this analysis. The statistically significant downregulated genes in the cell lines strongly inhibited by CM from the induced-ASCs from each organ were pooled with the genes that were mutated exclusively in the strongly inhibited cell lines and used to probe the Reactome Pathway Database V53^33,34^.

To determine correlation between the presence of mutated genes and the POC in the 3D-spheroid proliferation assay, an averaged POC together with its standard deviation was calculated by taking the mean of the POCs in each of the cell lines in which each mutated gene was present. Likewise, an averaged POC together with its standard deviation was calculated by taking the mean of the POCs in each of the cell lines in which each mutated gene was not present. P-values were calculated for each mutated gene. Log effect was calculated by subtracting the logarithm of the averaged POC in the cell lines without the mutation from the logarithm of the averaged POC in the cell lines with the mutation. Default settings were utilized when using the Gene Functional Classification Tool from the DAVID Bioinformatics Database^40,41^.

### Evaluation of MDA-MB-231 Cell Growth Using RealTime-GLO™ Reagent

MDA-MB-231 cells were seeded (500 cells/well in DMEM high-glucose supplemented with 10% FBS, L-glutamine and gentamycin) in black-walled 96 well plates with clear bottoms. 24 hours after cell seeding, CM (prepared as above) was supplemented with 2x NanoLuc Enzyme and 2x MT cell viability substrate per manufacturer’s protocol (RealTime-GLO™ MT Cell Viability Assay by Promega). Medium was filtered through a surfactant-free cellulose acetate 0.2 µm syringe filter (Sartorius) to eliminate precipitates. Filtered CM was diluted 1:1 with unfiltered CM and supplemented with 5% FBS and 2 mM glutamine. Cells were washed with PBS and exposed either to CM from induced or non-induced-ASCs or to regular MDA-MB-231 growth medium (DMEM high glucose supplemented with 5% FBS). Cell viability (emitted luminescence) measurements began 4 hours after the addition of all components to wells (to allow for equilibration of the assay). Luminescence was measured every 4 hours for 20 hours using Infinite F200PRO (Tecan). The experiment was repeated 4 times using three different preparations of induced-ASCs and two different preparations of non-induced-ASCs.

### Co-culture experiments of ASCs and MDA-MB-231 cells

MDA-MB-231 cells were seeded (3 × 10^4^ cells/well) in 24 well plates. Induced (n = 3 batches) or non-induced (n = 2 batches) ASCs were seeded on top of 5 µm pore Transwell inserts (3 × 10^4^ cells/Transwell) which pores do not enable ASC migration. Cells were seeded in DMEM high glucose medium containing 2 mM glutamine and 5% FBS separately. After 24 hours, Transwells seeded with ASCs were placed on top of wells seeded with MDA-MB-231 cells. CM was collected for Luminex assays after 24 and 48 hours of co-culture. CM was tested for the expression of the same cytokines as detailed above. Statistical analysis was conducted using paired sample t-test.

### Animal Care

7–8-week old female athymic Foxn1^nu^ nude mice (Envigo RMS, Israel) were housed in IVC cages in a dedicated HVAC animal facility at 22 ± 2 °C and relative humidity of 55 ± 15%. Mice were provided a commercial rodent diet *ad libitum* and allowed free access to autoclaved water. Animal studies were conducted at Science in Action (Ness Ziona, Israel), approved by the Council of Experiments on Animal Subjects of the Ministry of Health for the State of Israel (ethics approval nos. IL-15-10-316 and IL-16-07-213), and were carried out in accordance with all relevant Law and Regulations, which are based on the internationally recognized principles of the 3Rs, as defined in the Israeli Animal Welfare Law (1994).

### Xenograft Breast Cancer Models

In the ectopic study, 7-week old mice were injected subcutaneously (SubQ) in the right flank with 3 × 10^6^ MDA-MB-231 cells. Electronic calipers were used to measure tumor volumes weekly. On day 9, mice were randomized to minimize intragroup tumor volume range and intergroup mean tumor volume. A single intramuscular (IM) injection of ASCs in a volume of 50 μL was made into the right thigh muscle of each treated animal. Mice in the induced-ASC group were injected with 1 × 10^6^ ASCs on day 9 and 5 × 10^6^ ASCs on day 28, whereas mice in the non-induced-ASC group were injected with 5 × 10^6^ ASCs on day 28. Control mice were untreated.

In the orthotopic study, 8-week old mice were injected into the left inguinal (4^th^) mammary fat pad with 3 × 10^6^ cells of MDA-MB-231. Tumor volume and body mass determinations were conducted as described above. On day 6, mice were randomized as described above. Mice in the vehicle group were injected with PlasmaLyte weekly from day 6 to day 83, whereas mice in the induced-ASC group were injected with PlasmaLyte weekly from day 6 to day 41 and subsequently with induced-ASCs weekly from day 48 to day 83. All IM injections contained 1 × 10^6^ induced-ASC cells in 50 μL and were made in the left upper thigh muscle. On day 84, mice were sacrificed. Tumors, lymph nodes and lungs were removed for histological analyses.

### Histopathology

Histopathology was conducted at Smart Assays (Ness Ziona, Israel). Tissue samples were immersion-fixed in buffered formalin and processed for paraffin embedding. Paraffin blocks of tumor samples were prepared to allow for 5 μm sections.

#### Tumor necrosis

Tumor sections were stained with H&E for general histopathological and necrosis assessment. Tumors were graded for necrosis level based on the following scale: Grade 0: 0–10%, Grade 1: 10–25%, Grade 2: 25–50%, Grade 3: 50–75%, Grade 4: >75%.

#### Tumor cell proliferation

Sections were deparaffinized and subjected to heat-induced antigen retrieval (HIER) by boiling for 20 min in 0.05% citraconic anhydride solution, pH 7.4. Sections were incubated for 1 hour at RT with rabbit monoclonal antibody to Ki67 (clone SP6; Abcam Cat# ab16667), diluted 1:200 in TBST, washed in TBST and incubated for 30 min with horseradish peroxidase (HRP)-labeled anti-rabbit IgG (ZytoMed, Cat# ZUC032-10). HRP activity was assayed by incubation for 3 min in TIBS buffer containing 0.05% diaminobenzidine (DAB × 4HCl [Sigma; Cat# 32750]) and 0.015% hydrogen peroxide. Slides counterstained with hematoxylin were photographed using an Olympus BX-50 microscope equipped with a QuantiFire XI CCD camera (Optronics) coupled with an RGB tunable imaging filter (CRI). 3–5 non-overlapping images were obtained per sample (20x objective). Using ImageJ, images were subjected to background subtraction and color deconvolution, resulting in separation of the image into blue and brown monochrome images showing all nuclei and Ki67-immunostained nuclei, respectively. The percentage of the Ki67 labeled nuclei in all images related to the same sample was used as the proliferation index.

#### Tumor cell apoptosis

Sections were deparaffinized and subjected to HIER by boiling in 10 mM citric buffer (pH 6.0) for 20 min. After cooling, sections were incubated for 1 hour at RT with rabbit monoclonal antibody to active caspase 3 (clone E83-77; Abcam Cat# ab32042) diluted in 1:1000 in TBST. Incubation, detection and counterstaining were performed as described above. To quantify apoptosis, sections of immunostained cells were manually counted in 6–10 high power (x40 objective) microscopic fields.

#### Tumor vascularization

Sections were immunostained with rabbit monoclonal antibody to mouse CD34 (clone EP373Y, Abcam; Cat# ab81289), diluted 1:1000 to visualize endothelial cells. Immunostaining was performed as described above. The zone of the highest density of CD34+ cells was photographed in each section. Using ImageJ, the number of pixels was measured. The percentage of the immunostained area was calculated in relation to the image size (4.19 Mpi).

#### Lung metastases

Paraffin blocks with lung specimens were subjected to exhaustive systematic sectioning. IHC staining with rabbit monoclonal anti-cytokeratin 18 (CK18) antibody (clone EPR1626; Abcam, Cat# ab133263) diluted 1:600 was used to detect tumor cells in sections subjected to HIER in TE buffer pH 8.0. The rest of the IHC procedure was as described above.

#### Lymph node metastases

Each pair of lymph nodes (axillary, inguinal and lumbar) was embedded into one paraffin block and subjected to exhaustive systematic sectioning. Sections were immunostained with anti-CK18 antibody as described above.

### Serum Collection

Blood was collected from the orbital sinus on day 84 and allowed to clot for 15 min in serum separator tubes followed by centrifugation at 14,000 g for 10 min. Serum was aliquoted and kept in −80 °C until assayed by Luminex for the expression of the following murine cytokines: Adiponectin, G-CSF, IFN-β, IFN-γ, IL-1β, IL-2, IL-6, IL-10, IL-12p70, IL-15, II-17A, IL-18, IL-23, IL-28, MIP-1α, MMP-1, MMP-2, OPN, sICAM-1, stromal cell-derived factor 1 alpha (SDF-1α), TNF-α, TGF-β1/2/3 and VEGF. Statistical analysis was performed with one way ANOVA using the Duncan *post hoc* test. Outliers that were >(Q3 + 1.5 × IQR) or < (Q1 − 1.5 × IQR) were removed prior to performing statistical analysis. (Q1 is first quartile, Q3 is third quartile and IQR is interquartile range).

### Data availability

The datasets generated during and/or analyzed during the current study are available from the corresponding author upon reasonable request. Pluristem Ltd. is a for-profit company that has obligations to its shareholders; therefore, materials will be released only by a material transfer agreement. All reasonable requests should be made to the corresponding author.

## Electronic supplementary material

Supplementary Information

Supplementary Dataset S1
