# Supplementary material for: Human Placental-Derived Adherent Stromal Cells Co-Induced with TNF-α and IFN-γ Inhibit Triple-Negative Breast Cancer in Nude Mouse Xenograft Models
Source: Sci Rep. 2018 Jan 12;8:670. doi: 10.1038/s41598-017-18428-1 (PMC5766494; doi:10.1038/s41598-017-18428-1)
Supplement: Supplementary file 1 — Supplementary Information [file 41598_2017_18428_MOESM1_ESM.pdf]

# Supplementary Information

# Human Placental-Derived Adherent Stromal Cells Co-Induced with TNF-α and IFN-γ Inhibit Triple-Negative Breast Cancer in Nude Mouse Xenograft Models

Hoshea Allen[1‡], Niva Shraga-Heled[1‡], Michal Blumenfeld[1], Tamar Dego-Ashto[1], Dana Fuchs-Telem[1], Ariel Gilert[1], Zami Aberman[1], and Racheli Ofir[1*]

[1] Pluristem Ltd., Haifa, Israel, 31905

‡ H.A. and N.S.-H. contributed equally to this work

* Correspondence should be addressed to R.O. (email: racheli@pluristem.com)

## Supplementary Table S1: Time-Dependent Concentration of Cytokines in the Conditioned Medium of TNF-α/IFN-γ-Induced Human Placental-Derived ASCs

| Cytokine | Days from Seeding | | | | | | Non-Induced ASCs |
|---|---|---|---|---|---|---|---|
| | 2 | 5 | 7 | 9 | 12 | 14 | |
| CXCL8 | 16,887 ± 9,069 | 3,723 ± 2,644 | 4,546 ± 547 | 681 ± 71 | 2,268 ± 144 | 1,151 ± 239 | 313 |
| CCL2 | 2,082 ± 448 | 1,109 ± 491 | 522 ± 241 | 281 ± 134 | 362 ± 168 | 1,330 ± 872 | 98 |
| CCL5 | 16,549 ± 7,105 | 3,399 ± 755 | 627 ± 166 | 139 ± 46 | 27 ± 10 | 15 ± 02 | 0 |
| GM-CSF | 365 ± 107 | 28 ± 7 | 41 ± 10 | 08 ± 02 | 16 ± 03 | 19 ± 03 | 3 |
| G-CSF | 1,501 ± 358 | 40 ± 5 | 2 ± 2 | 2 ± 2 | 4 ± 4 | 5 ± 5 | 0 |
| IL-6 | 5,604 ± 1,609 | 341 ± 94 | 203 ± 9 | 49 ± 6 | 79 ± 1 | 117 ± 12 | 8 |
| CXCL10 | 4,941 ± 963 | 245 ± 63 | 2 ± 1 | 2 ± 0 | 2 ± 1 | 5 ± 2 | 0 |
| IFN-γ | 366 ± 44 | 91 ± 11 | 47 ± 10 | 9 ± 2 | 13 ± 2 | 10 ± 1 | 8 |
| CXCL9 | 13,465 ± 2,632 | 4,163 ± 49 | 0 | 25 ± 7 | 0 | 0 | 0 |
| HGF | 133 ± 55 | 115 ± 45 | 16 ± 8 | 16 ± 9 | 22 ± 7 | 41 ± 22 | 158 |

## Supplementary Methods for Table S1

Induced-ASCs (3 different cell batches) were seeded $2x10^5$ cells/well in 6 well plates in DMEM low glucose supplemented with 2 mM L-glutamine, 10% FBS and 50 μg/ml gentamycin. Cells were passaged 5 and 9 days after seeding (when reaching 80-90% confluence). Two, five, seven, nine, 12 and 14 days from the day of seeding CM was collected from two wells as described, and cells were counted. Concentration of 10 highly expressed cytokines (CXCL8, CCL2, CCL5, GM-CSF, G-CSF, IL-6, CXCL10, IFN- γ, CXCL9 and HGF) were measured using custom made Luminex panels. Absolute concentrations as measured by Luminex in each well were divided by the number of cells and days since last medium renewal to calculate pg/$10^6$ cells/day. Concentrations are expressed as mean ± SEM (n=3).

Supplementary Figure S1: Interferon Signaling and MHC Class I Antigen Processing and Presentation Pathways Enriched in Key Gene Sets

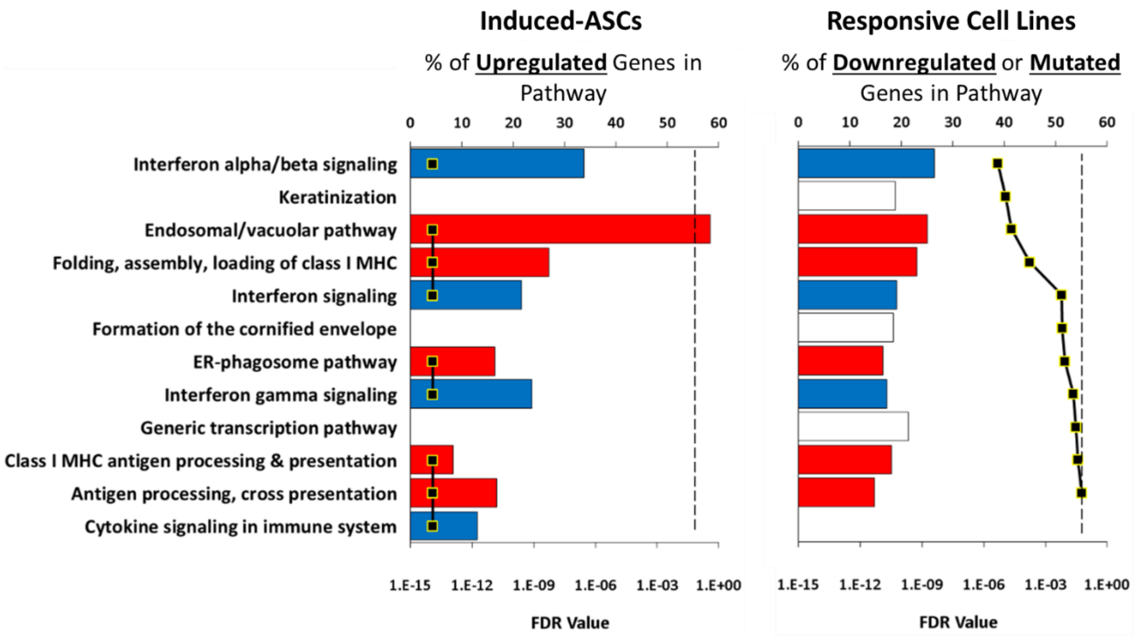


The most statistically significant pathways enriched in genes upregulated in TNF-$\alpha$/IFN-$\gamma$-induced placental-derived ASCs are shown on the left. Pathways enriched in genes downregulated and/or exclusively mutated in the responsive cell lines are shown on the right. Blue represents cytokine signaling in the immune system, and red represents MHC class I mediated antigen processing and presentation. The false discovery rate (FDR) value, the corrected enrichment probability for each pathway, is shown by the thick black line. Dashed vertical line represents FDR=0.05.

Supplementary Figure S2: Mutations in Transcription Factors and Protein Kinases Correlate with the Anti-Proliferative Response to CM from TNF-α/IFN-γ-Induced Placental-Derived ASCs

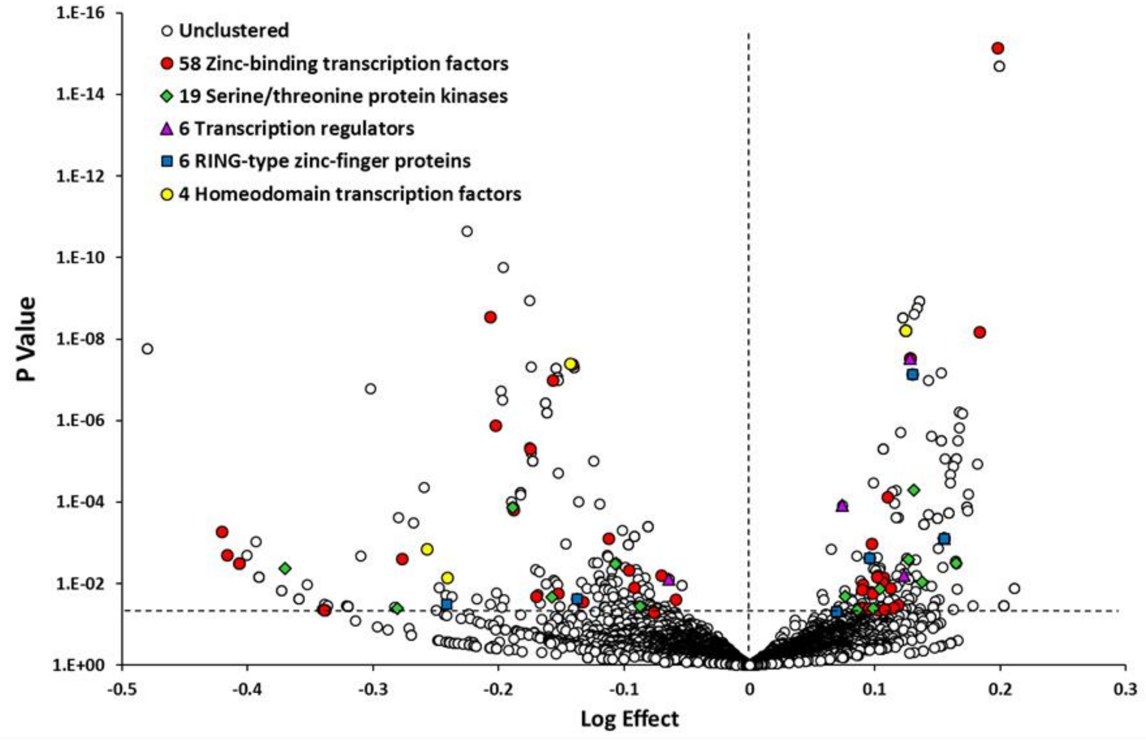


Mutation association plot showing somatic mutations in cancer cell lines that positively or negatively correlate with the anti-proliferative response to CM from induced placental-derived ASC. Each of the 6666 data points (whether colored or not) represent an individual mutated gene. Those with a positive log effect correlate with a pro-proliferative effect; those with a negative log effect correlate with an anti-proliferative effect. 611 genes appear above the horizontal dashed line, which represents p=0.05. The statistical significance of the five clusters found in these 611 genes are as follows: zinc-binding transcription factors (p=0.00013), serine/threonine protein kinases (p=0.0011), transcription regulators (p=0.0066), RING-type zinc-finger proteins (p=0.011), and homeodomain transcription factors (p=0.034).
